# Functional characterization in *Xenopus* oocytes of Na^+^ transport systems from durum wheat reveals diversity among two HKT1;4 transporters

**DOI:** 10.1093/jxb/ert361

**Published:** 2013-11-05

**Authors:** Siwar Ben Amar, Faiçal Brini, Hervé Sentenac, Khaled Masmoudi, Anne-Aliénor Véry

**Affiliations:** ^1^Plant Protection and Improvement Laboratory, Center of Biotechnology of Sfax (CBS)/University of Sfax, B.P. ‘1177’ 3018, Sfax, Tunisia; ^2^Biochimie & Physiologie Moléculaire des Plantes, UMR 5004 CNRS/ 386 INRA/SupAgro Montpellier/Université Montpellier 2, Campus SupAgro-INRA, 34060 Montpellier Cedex 2, France

**Keywords:** Durum wheat, electrophysiology, HKT1;4, salt tolerance, sodium transport, *Xenopus* oocyte.

## Abstract

Plant tolerance to salinity constraint involves complex and integrated functions including control of Na^+^ uptake, translocation, and compartmentalization. Several members of the high-affinity K^**+**^ transporter (HKT) family, which comprises plasma-membrane transporters permeable to K^**+**^ and Na^**+**^ or to Na^**+**^ only, have been shown to play major roles in plant Na^**+**^ and K^**+**^ homeostasis. Among them, HKT1;4 has been identified as corresponding to a quantitative trait locus (QTL) of salt tolerance in wheat but was not functionally characterized. Here, we isolated two *HKT1;4*-type cDNAs from a salt-tolerant durum wheat (*Triticum turgidum* L. subsp. *durum*) cultivar, Om Rabia3, and investigated the functional properties of the encoded transporters using a two-electrode voltage-clamp technique, after expression in *Xenopus* oocytes. Both transporters displayed high selectivity for Na^+^, their permeability to other monovalent cations (K^**+**^, Li^**+**^, Cs^**+**^, and Rb^**+**^) being ten times lower than that to Na^**+**^. Both TdHKT1;4-1 and TdHKT1;4-2 transported Na^+^ with low affinity, although the half-saturation of the conductance was observed at a Na^+^ concentration four times lower in TdHKT1;4-1 than in TdHKT1;4-2. External K^+^ did not inhibit Na^+^ transport through these transporters. Quinine slightly inhibited TdHKT1;4-2 but not TdHKT1;4-1. Overall, these data identified TdHKT1;4 transporters as new Na^+^-selective transporters within the HKT family, displaying their own functional features. Furthermore, they showed that important differences in affinity exist among durum wheat HKT1;4 transporters. This suggests that the salt tolerance QTL involving HKT1;4 may be at least in part explained by functional variability among wheat HKT1;4-type transporters.

## Abstract (precis)

*HKT1;4* was linked to a salt tolerance QTL in wheat. Here, two wheat HKT1;4-type transporters were functionally characterised in *Xenopus* oocytes. Beside shared properties, differences in Na^+^ transport affinity were evidenced. Such functional diversity sheds light on the QTL bases

## Introduction

High soil salinity, which limits the yield of most crop species, is a major constraint for agriculture, particularly affecting arid and semi-arid areas (Rengasamy, 2006; James *et al.*, 2012). Plant tolerance to salinity constraint involves complex and integrated responses, at the cell, metabolic, and even anatomic levels, the relative contribution of which is dependent on the plant species. However, it involves, in every species, the ability of the plant to maintain efficient root K^+^ uptake in the presence of high Na^+^ concentrations, and to tightly control net Na^+^ uptake by the roots and Na^+^ translocation and accumulation in leaves, as young leaves and photosynthetic tissues are very sensitive to salt stress (Apse and Blumwald, 2007; Munns and Tester, 2008).

Among the identified determinants of plant salt tolerance have been found several Na^+^ transport systems (Pardo *et al.*, 2006; Munns and Tester, 2008). The first transport systems that have been identified as playing a role in salt tolerance are H^+^/Na^+^ antiporters present either at the plasma membrane or at the tonoplast. The importance of the plasma-membrane Salt Overly Sensitive 1 (SOS1) antiporter in plant salt tolerance could be linked to the involvement of this system in the control of root-to-shoot Na^+^ transport and the maintenance of Na^+^/K^+^ homeostasis upon salt stress (Wu *et al.*, 1996; Shi *et al.*, 2002; Olías *et al.*, 2009). NHX vacuolar antiporters, which allow Na^+^ and K^+^ accumulation in the vacuole, are thought to play important roles in osmoregulation and to prevent overaccumulation of Na^+^ in the cell cytosol upon salt stress (Apse *et al.*, 1999, 2003; Brini *et al.*, 2007; Rodríguez-Rosales *et al.*, 2009). Another group of transport systems identified as key determinants of plant salinity tolerance are Na^+^ transporters from the so-called high-affinity K^+^ transporter’ (HKT) family (Munns and Tester, 2008; Hauser and Horie, 2010). In *Arabidopsis*, AtHKT1 has been shown to control Na^+^ accumulation in the shoots in salt-stress conditions by mediating Na^+^ retrieval from the ascending xylem sap in the roots (Sunarpi *et al.*, 2005; Davenport *et al.*, 2007) and Na^+^ recirculation from shoots to roots via phloem sap loading (Berthomieu *et al.*, 2003). Analysis of several quantitative trait loci (QTLs) of salt tolerance in rice and wheat has provided further evidence for the importance of *HKT* genes in control of Na^+^ exclusion from the leaves upon salinity stress (Ren *et al.*, 2005; Huang *et al.*, 2006; Byrt *et al.*, 2007; James *et al.*, 2011).

Whereas the HKT family is comprised of a single gene in *Arabidopsis thaliana*, encoding a Na^+^-selective transporter (Uozumi *et al.*, 2000; Mäser *et al.*, 2001), it is much larger in cereals: nine *HKT* genes are present in rice (*Oryza sativa*), and six to twenty-four *HKT* are expected from Southern blot analyses to be present in barley (*Hordeum vulgare*) and wheat species with different ploidy levels (Garciadeblás *et al.*, 2003; Huang *et al.*, 2008). Partial characterization of the rice HKT family has revealed an important diversity, at both the expression and functional levels, suggesting that the different members have specific roles (Corratgé-Faillie *et al.*, 2010). Some members were indeed reported to display specific expression in vascular tissues (Ren *et al.*, 2005), while other members were rather/also shown to be expressed in other tissues, for instance in root peripheral layers (Kader *et al.*, 2006; Horie *et al.*, 2007; Jabnoune *et al.*, 2009). At the functional level, strong differences in relative permeability to Na^+^ and K^+^ were observed between rice HKT members, some of them being Na^+^ selective, like AtHKT1, while others displayed high permeability to both Na^+^ and K^+^ or high permeability to K^+^ (Horie *et al.*, 2001; Ren *et al.*, 2005; Jabnoune *et al.*, 2009; Horie *et al.*, 2011; Oomen *et al.*, 2012; Sassi *et al.*, 2012). Differences in Na^+^/K^+^ permeability correlated well with overall sequence differences, which led to the sorting of HKT transporters into two subfamilies corresponding to the two main phylogenetic branches of the rice family: subfamily 1 regroups Na^+^-selective HKT transporters, and subfamily 2 comprises transporters displaying permeability to K^+^ (Platten *et al.*, 2006). In addition to the relative permeability to Na^+^ and K^+^, strong functional differences between rice HKT members were also shown to concern Na^+^ transport affinity (Garciadeblás *et al.*, 2003; Jabnoune *et al.*, 2009).

*HKT* genes shown to be associated to QTLs of salt tolerance in rice and wheat belong to subfamily 1. In rice, the *SKC1* trait of leaf K^+^ homeostasis upon salt stress, identified in a population derived from a cross between the salt-tolerant cultivar Nona Bokra and the salt-sensitive one Koshihikari, corresponds to the *HKT1;5* gene (Lin *et al.*, 2004; Ren *et al.*, 2005). *OsHKT1;5* was shown to encode a Na^+^-selective transporter, insensitive to external K^+^ in both Nona Bokra and Koshihikari. The Nona Bokra’s variant of the transporter was found to be slightly more conductive to Na^+^ than the Koshihikari’s one (Ren *et al.*, 2005). *OsHKT1;5* transcripts were localized in vascular tissues, mainly in root xylem parenchyma, which suggested a role for OsHKT1;5 in Na^+^ retrieval from the xylem sap upon salt stress (Ren *et al.*, 2005). In wheat, *HKT1;5*-like genes have also been found to correspond to QTLs of Na^+^ exclusion from the shoots upon salt stress (Dvořák *et al.*, 1994; Dubcovsky *et al.*, 1996; Byrt *et al.*, 2007). Durum wheat (*Triticum turgidum* L. subsp. *durum*; tetraploid, comprised genomes A and B) is more salt sensitive than bread wheat (*Triticum aestivum*; hexaploid, comprised genomes A, B, and D) and the wheat ancestor *Triticum monococcum* (diploid, possessing an A^m^ genome), and displays a poorer ability to exclude Na^+^ from the leaf blades (Gorham *et al.*, 1990). A locus enabling improvement of leaf Na^+^ exclusion in durum wheat, *Kna1*, found in the D genome of bread wheat, corresponds to an *HKT1;5*-like gene sharing 66% identity with the rice *HKT1;5* gene (Byrt *et al.*, 2007). Another source of leaf Na^+^ exclusion, *Nax2*, absent in durum or bread wheat but found in the A genome of the diploid wheat ancestor *T. monococcum*, also corresponds to an *HKT1;5*-like gene sharing 94% identity with that from the bread wheat D genome (James *et al.*, 2006; Byrt *et al.*, 2007). All these different *HKT1;5* genes associated with QTLs of salt tolerance in rice and wheat share strong expression in roots and play a role in the control of root-to-shoot Na^+^ transfer, probably via retrieval of Na^+^ from the xylem sap in the roots (Ren *et al.*, 2005; James *et al.*, 2006; Byrt *et al.*, 2007).

In wheat, a slightly different trait of Na^+^ exclusion from the shoots upon salt stress, *Nax1*, introgressed from *T. monococcum* into a durum wheat cultivar, was shown to concern another *HKT* gene from subfamily 1, *HKT1;4* (Lindsay *et al.*, 2004; James *et al.*, 2006; Huang *et al.*, 2006). The *Nax1* locus is indeed associated with the exclusion of Na^+^ from leaf blades only upon salt stress, both by retrieval of Na^+^ from the xylem in roots and leaf sheaths and by recirculation of Na^+^ from the shoots to the roots via the phloem (James *et al.*, 2006). In spite of its importance in wheat salt tolerance, HKT1;4, in contrast to HKT1;5, was not functionally characterized, in either wheat or rice. Here, we isolated two *HKT1;4*-type isoforms from a salt-tolerant durum wheat cultivar and investigated the functional properties of the two transporters using *Xenopus laevis* oocytes as a heterologous expression system.

## Materials and methods

### Plant material and growth conditions

Om Rabia3, a salt-tolerant Tunisian cultivar of durum wheat [*T. turgidum* L. subsp. *durum* (Desf.) Husn.)], was supplied by the Laboratoire de Physiologie Végétale from the INRAT (Tunis, Tunisia). Seeds were surface sterilized in 0.5% NaOCl for 15min, then washed three times with sterile water and placed on a sheet of Whatman #1 filter paper in Petri dishes in the dark for germination. Four-day-old seedlings were transferred to a hydroponics system in half-strength Hoagland’s solution (Epstein, 1972) and incubated at 25 °C in a growth chamber under a 16h light/8h dark photoperiod and 60±10% relative humidity. A salt treatment by addition of 50mM NaCl for 48h was performed when plants reached the third-leaf stage.

### Cloning of *HKT1;4* durum wheat isoforms

Total RNA from durum wheat leaf was extracted using an RNeasy Mini kit (Qiagen). To remove contaminating genomic DNA, total RNA (10 µg) was treated with DNase (Promega). DNase-treated RNA samples (0.5 µg) were reverse transcribed using Moloney murine leukemia virus reverse transcriptase (Invitogen). The reverse transcription (RT) reaction was performed at 37 °C for 1h using 2 µM oligo-(dT)_18_ primer. Two microlitres of first-strand cDNA was used as template for PCR amplification of the *HKT1;4* isoforms, using *Pfu* polymerase in a reaction solution prepared according to the instructions of the manufacturer (Fermentas). Primers, designed on the basis of the sequence of the *T. monococcum HKT1;4*-*A2* gene were 5′-ATGGCCGGAGCTCATCATAAG-3′ (forward) and 5′-CTAACTAAGCTTCCAGGCTTTG-3′ (reverse). The amplification protocol, after denaturation of the cDNA for 5min at 94 °C, consisted of 35 cycles of 30 s at 94 °C, 30 s at 60 °C, and 90 s at 72 °C, with a final extension for 5min at 72 °C. Amplified products were cloned using the pGEM-T Easy vector system (Promega), and successful isolation of *HKT1;4*-type cDNA was confirmed by digestion and sequencing.

Verification of 5′ and 3′ ends of cloned cDNA was performed by 5′- and 3′-rapid amplification of cDNA ends (RACE) on DNase-treated RNA samples using a FirstChoice RLM Race kit (Ambion). Primers hybridizing specifically to *TdHKT1;4-1* or *TdHKT1;4-2* used for 5′-RACE were 5′-ATGCGATGACAGGAGGGACAATGC-3′ (hybridization at +99 nt from the start of the *TdHKT1;4-1* coding sequence) and 5′-CATGAGGGACATTACATTGTTGAGCG-3′ (hybridization at +90bp from the start of the *TdHKT1;4-2* coding sequence). Those used for 3′-RACE, were 5′-GGCGTCAAGGACCAACCCAG-3′ (hybridization at +1315 nt in *TdHKT1;4-1* cDNA) and 5′-CCAGCGTGAAGGACCATCCCA-3′ (hybridization at +1307 nt in *TdHKT1;4-2* cDNA), respectively.

Correction of the cloned *TdHKT1;4-2* cDNA sequence, following 5′-RACE results, was performed using a QuikChange Site-Directed Mutagenesis kit (Stratagene). The following forward and reverse primers introducing a single base-pair mutation were used for mutagenesis: 5′-GGCCGGAGCTCATCGTAAGGTCCGCGAGC-3′ and 5′-CGTCGCGGACCTTACGATGAGCTCCGGCC-3′.

### Expression in *X. laevis* oocytes

The coding regions of *TdHKT1;4-1* and *TdHKT1;4-2* were subcloned into the pGEMXho vector (derived from pGEMDG; D. Becker, Würzburg) downstream from the T7 promoter and between the 5′- and 3′-untranslated regions of the *Xenopus β-globin* gene. Capped and polyadenylated copy RNA (cRNA) were synthesised *in vitro*, from linearized vector (for *TdHKT1;4-1*) or from high-fidelity PCR-amplified (using iProof polymerase; Biorad) T7 promoter to the linearization site fragment (for *TdHKT1;4-2*), using an mMESSAGE mMACHINE T7 kit (Ambion). Oocytes, isolated as described previously (Véry *et al.*, 1995), were injected with either 20ng of *TdHKT1;4-1* or *TdHKT1;4-2* cRNA or 20 nl of diethylpyrocarbonate-treated water for control oocytes, and then kept at 18 °C in ND96 solution (96mM NaCl, 2mM KCl, 1.8mM CaCl_2_, 1mM MgCl_2_, 2.5mM sodium pyruvate, and 5mM HEPES/NaOH, pH 7,4) supplemented with 0.5mg l^–1^ of gentamicin, until electrophysiological recordings.

### Two-electrode voltage-clamp method

Whole-oocyte currents were recorded using a two-electrode voltage-clamp technique 1–2 d after cRNA injection, as described by Mian *et al.* (2011). All electrodes were filled with 3M KCl. The external solution bathing the oocyte was continuously percolated during the voltage-clamp experiment. All bath solutions contained a background of 6mM MgCl_2_, 1.8mM CaCl_2_, and 10mM MES-1,3-bis[tris(hydroxymethyl)methylamino]propane, pH 5.5. Monovalent cations were added to the background solution as glutamate or chloride salts. The chloride concentration was constant, except when otherwise mentioned, in each set of solutions. d-Mannitol was added when necessary to adjust the osmolarity, which was set to 220–240 mosM in each set of solutions, except in experiments aimed at examining the effect of osmolarity variation in the range 150–400 mosM. The actual concentrations of K^+^ and Na^+^ in the bath solutions were checked systematically by flame photometry. To extract HKT-mediated currents from total oocyte currents, mean currents recorded in water-injected control oocytes from the same batch in the same ionic conditions were subtracted from those recorded in HKT-expressing oocytes. TdHKT1;4-1 and TdHKT1;4-2 current–voltage (I–V) relationships were constructed with transporter-extracted currents.

## Results

### Isolation of two *HKT1;4* durum wheat genes

**Fig. 1.**
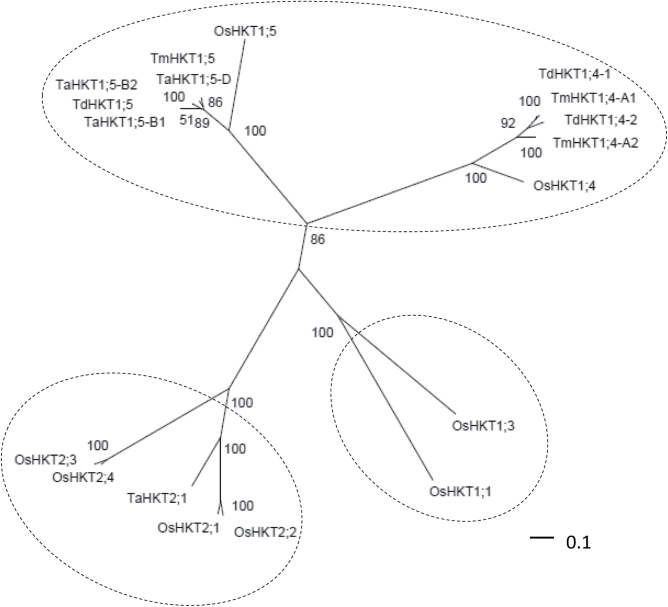
Phylogenetic relationships between HKT transporters in rice and wheat. The unrooted phylogenetic tree was constructed using full polypeptide sequences aligned with MUSCLE (http://www.bioinformatics.nl/tools/muscle.html; Dereeper *et al.*, 2008), and the neighbour-joining method with 1000 bootstrap replicates, using PhyML software (http://phylogeny.lirmm.fr). The tree was drawn using Dendroscope (Huson *et al.*, 2007). Bootstrap values (as percentages) are indicated at the corresponding nodes. The protein accession numbers are: OsHKT1;1, Q7XPF8.2; OsHKT1;3, Q6H501.1; OsHKT1;4, Q7XPF7.2; OsHKT1;5, Q0JNB6.1; OsHKT2;1, Q0D9S3.1; OsHKT2;2, BAB61791.1; OsHKT2;3, Q8L481.1; OsHKT2;4, Q8L4K5.1; TaHKT1;5-B1, ABG33943; TaHKT1;5-B2, ABG33944; TaHKT1;5-D, ABG33945; TaHKT2;1, AAA52749; TmHKT1;4-A1, ABK41858; TmHKT1;4-A2, ABK41857; TmHKT1;5, ABG33939; TdHKT1;4-1, KF443078; TdHKT1;4-2, KF443079. Os, *Oryza sativa*; Ta, *Triticum aestivum*; Td, *Triticum turgidum* subsp. *durum*; Tm, *Triticum monococcum*.

In durum wheat, analysis by Southern blotting has suggested the existence of five *HKT1;4-*like genes (Huang *et al.*, 2008). Isolation of durum wheat *HKT1;4* cDNA was attempted, taking advantage of the availability of two full-length *HKT1;4* gene sequences in the wheat ancestor *T. monococcum* (Fig. 1). The coding regions of the two *T. monococcum* genes, *TmHKT1;4*-*A1* and *TmHKT1;4*-*A2*, share 88% identity and display, at the start and end, strong sequence identity. This encouraged us to use the *TmHKT1;4*-*A1*/*A2* sequence to design primers for the cloning by reverse transcription-PCR of durum wheat *HKT1;4* cDNA. Two durum wheat *HKT1;4* full-length cDNA were isolated by this strategy. Their genome origin (i.e. A or B) being unknown, they were named *TdHKT1;4-1* and *TdHKT1;4-2* (GenBank accession nos KF443078 and KF443079, respectively). The start and end sequences of the two clones, imposed by the primers used, were verified by 5′ and 3′ RACE. A mutation at position 17 from ATG was detected in *TdHKT1;4-2* and was corrected by site-directed mutagenesis.

*TdHKT1;4-1* and *TdHKT1;4-2* presented open reading frames of 1692 and 1686bp, respectively, sharing 92% identity. At the amino acid level, TdHKT1;4-1 and TdHKT1;4-2 thus comprised 564 and 562 residues, respectively, and displayed 89% identity. This level of identity between the two durum wheat transporters suggested that they are not encoded by different alleles of the same gene but rather by two different *HKT1;4*-type genes. The phylogenetic relationships were analysed between the two durum wheat transporters, the rice HKT transporters, and the other HKT members already identified in wheat. Three branches were clearly distinguishable in the resulting phylogenetic tree (Fig. 1), corresponding to the HKT subfamily 2 and two distinct groups within subfamily 1, one comprising HKT1;1 and HKT1;3, and the other comprising HKT1;4 and HKT1;5. The two identified durum wheat transporters were clearly shown to be related to HKT1;4 members. They display ∼65% identity with OsHKT1;4. As expected, both were close to the HKT1;4-like transporters already identified in *T. monococcum*, and particularly to TmHKT1;4-A1 (Fig. 1): TdHKT1;4-1 was very close to TmHKT1;4-A1, sharing the same length and 97% identity, TdHKT1;4-2 was 2 aa shorter and shared 88% identity with TmHKT1;4-A1. Both durum wheat HKT1;4-like transporters shared 84% identity with TmHKT1;4-A2 and were slightly longer (by 7–9 aa).

#### *TdHKT1;4-1* and *TdHKT1;4-2* expressed in *Xenopus* oocytes mediate Na^+^-selective transport

**Fig. 2.**
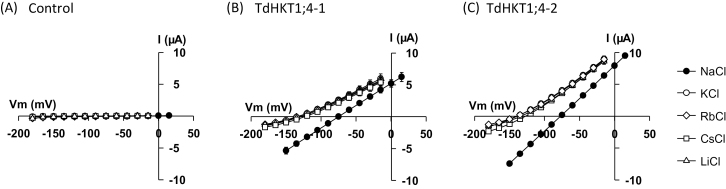
TdHKT1;4-1 and TdHKT1;4-2 function as monovalent cation transporters with a strong preference for Na^+^ in *Xenopus* oocytes. Bath solutions contained the standard background supplemented with NaCl, KCl, RbCl, CsCl, or LiCl, at 3mM. The voltage-clamp protocol consisted of 12 pulses of 1 s, with a voltage increment of 15 mV between pulses. (A) Currents from control (H_2_O-injected) oocytes plotted against applied voltages. (B, C) Currents flowing through TdHKT1;4-1 (B) and TdHKT1;4-2 (C) transporters versus applied voltages. Data are means±standard error (SE) (*n*=4) and are representative of five experiments performed on different oocyte batches.

TdHKT1;4-1 and TdHKT1;4-2 transport properties were studied by performing voltage-clamp experiments in *Xenopus* oocytes. As TdHKT1;4-1 and TdHKT1;4-2 both belong to HKT subfamily 1, they were expected to be Na^+^ selective. For both systems, injection of 20ng of transporter cRNA in oocytes resulted in large exogenous currents in Na^+^-containing solutions, from 1 d after injection: for example, there was about a 50- and 75-fold increase in oocyte conductance in the presence of 3mM NaCl upon expression of TdHKT1;4-1 and TdHKT1;4-2, respectively (Fig. 2). This indicated that both transporters were efficiently expressed and targeted to the oocyte membrane.

To investigate the cation selectivity of both durum wheat HKT1;4 transporters, oocyte currents were elicited in the presence of different monovalent cations (Na^+^, K^+^, Rb^+^, Cs^+^, and Li^**+**^) at a concentration of 3mM (Fig. 2). The membrane conductance in HKT1;4-expressing oocytes (Fig. 2B, C) was in all solutions at least five times higher than that in water-injected control oocytes (Fig. 2A), allowing precise determination of the HKT transporter cation selectivity. In the presence of 3mM external Na^+^, TdHKT1;4-1 and TdHKT1;4-2 mediated large inward and outward currents reversing close to –75 mV (Fig. 2B, C). When another monovalent cation (K^+^, Rb^+^, Cs^+^, or Li^+^) replaced Na^+^ at the same concentration, both inward and outward currents could still be observed, but the I–V relationship was shifted negatively by 55–60 mV in both transporters (Fig. 2B, C). Such shifts in I–V relationships and highly negative membrane polarizations were not observed in control oocytes, in which the recorded resting membrane potentials were never more negative than –50 mV and the shifts in I–V relationships upon changing the cation in the bath were lower than 15 mV (Fig. 2A). Thus, expression of TdHKT1;4-1 or TdHKT1;4-2 gave rise to exogenous conducting pathways displaying selectivity for Na^+^. In oocytes expressing either of the two HKT1;4-type transporters, replacing Na^+^ by K^+^, Rb^+^, Cs^+^, or Li^+^ in the bath also reduced (by about three times) the inward conductance of the transporter (Fig. 2B, C). As a whole, these experiments indicated that TdHKT1;4-1 and TdHKT1;4-2 are both Na^+^-selective, as expected. Besides Na^+^, which was the most permeant cation, the other tested monovalents permeated through these two systems very similarly. The level of discrimination between Na^+^ and the other monovalents was strongly similar in the two transporters.

### Affinity for Na^+^ of the two durum wheat HKT1;4 transporters

**Fig. 3.**
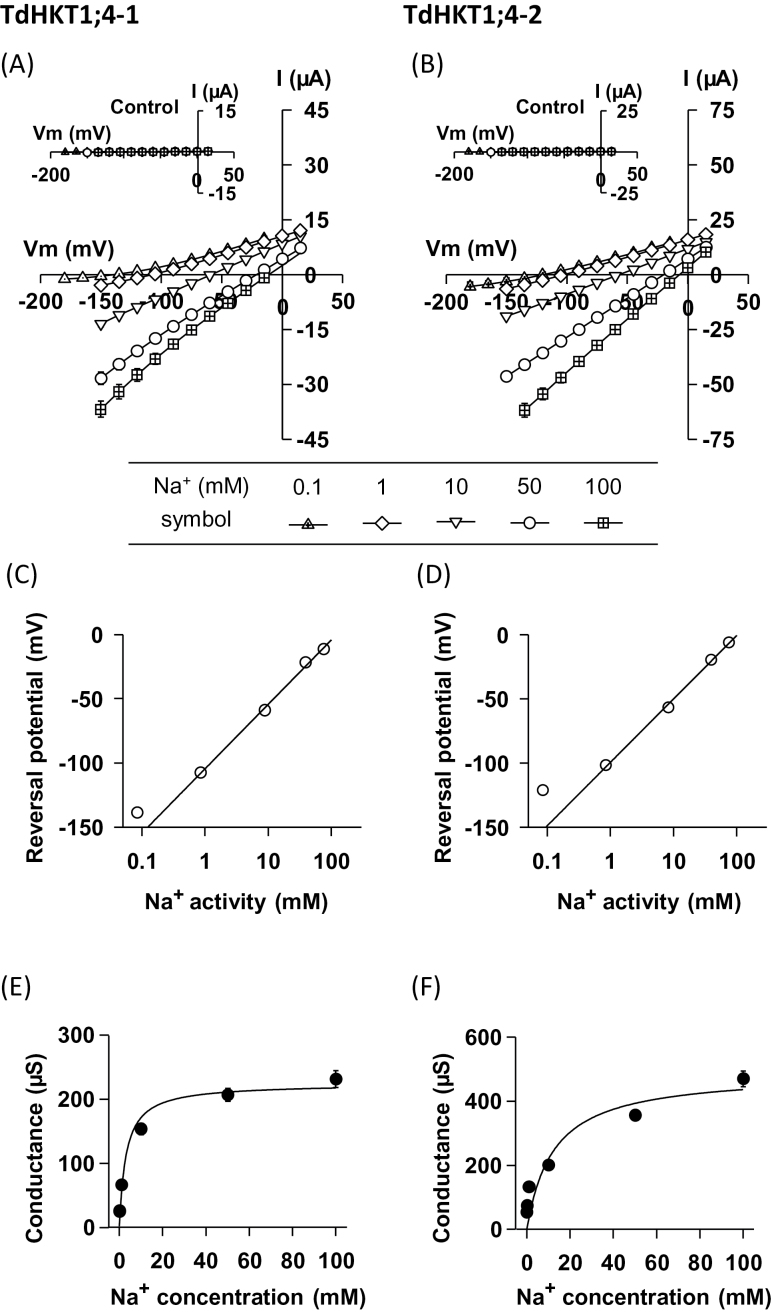
TdHKT1;4-1 and TdHKT1;4-2 transporters differ in their affinity for Na^+^. (A, B) Currents flowing through TdHKT1;4-1 (A) or TdHKT1;4-2 (B) transporters versus applied voltages in the presence of varying external Na^+^ concentrations (0.1, 1, 10, 50, and 100mM). Na^+^ was provided as glutamate salt. Data are means±SE (*n*=4 in A and *n*=5 in B) and are representative of six experiments performed on different oocyte batches. Insets in (A) and (B): I–V relationships in water-injected oocytes belonging to the same batch as HKT-expressing oocytes. Experimental conditions were the same as for HKT-expressing oocytes. Currents are means±SE (*n*=4 in A and *n*=3 in B). (C, D) Zero-current potentials through TdHKT1;4-1 (C) and TdHKT1;4-2 (D) versus bath Na^+^ activity. Current reversal potentials were obtained from the I–V data shown in (A) and (B). (E, F) Variation of TdHKT1;4-1 (E) and TdHKT1;4-2 (F) macroscopic inward conductance with external Na^+^ concentration. Macroscopic inward conductances were defined as slopes of I–V relationships between the three most negative imposed potentials in each ionic condition. The conductances in (E) and (F) were extracted from the I–V data shown in (A) and (B). Inward conductances plotted against external Na^+^ concentrations were fitted (solid line) with a Michaelis–Menten equation to determine the apparent half-saturation constant (*K*_M_). Fitted parameters were as follows: *K*_M_ ∼3mM and *G*_max_ (maximum whole-cell conductance) ~225 µS (E); *K*_M_ ~12mM and *G*_max_ ∼490 µS (F).

Na^+^ transport through TdHKT1;4-1 and TdHKT1;4-2 was analysed further by exposing the oocytes to bath solutions containing varying concentrations of Na^+^ (0.1, 1, 10, 50, and 100mM). In water-injected control oocytes, currents were of very low amplitude in all these solutions (Fig. 3A, B, inset) and responded weakly to Na^+^ concentration changes: there was an increase of only ∼2-fold of the oocyte inward conductance and shift of the resting membrane potential by ∼25 mV upon a Na^+^ increase from 0.1 to 100mM (not shown). Oocytes expressing TdHKT1;4-1 or TdHKT1;4-2, in contrast, displayed currents that were much larger than those in water-injected oocytes (conductance 15 to >100 times greater), and that strongly responded to changes in external Na^+^ concentration in the tested range of concentrations (Fig. 3A, B): for example, there was a 9- and 6-fold increase of the oocyte inward conductance and a mean shift of the resting membrane potential by 127 and 115 mV, upon a Na^+^ increase from 0.1 to 100mM, in TdHKT1;4-1- and TdHKT1;4-2-expressing oocytes, respectively.

Detailed analysis of the currents passing through each of the two transporters indicated that the zero-current potential varied linearly with the logarithm of the external activity of Na^+^ within the 1–100mM concentration range (Fig. 3C, D). The slope of the zero-current potential variation was of 50 mV per 10-fold increase in Na^+^ activity in both transporters, which was close to the expected value (58 mV per 10-fold activity increase) in a purely Na^+^-selective system mediating passive transport. At submillimolar Na^+^ concentrations, the slope was lower, which probably reflects a slight permeability of both systems to (an)other ion(s) present in the bath solution. Overall, this indicated that TdHKT1;4-1 and TdHKT1;4-2 behave as typical Na^+^-selective ‘uniporters’ from the HKT subfamily 1 (Jabnoune *et al.*, 2009).

The increase in both transporters’ inward conductance with increasing Na^+^ concentration became saturated at high Na^+^ concentration. Fitting the inward conductance versus the external Na^+^ relationship with Michaelis–Menten hyperbolic functions allowed to compare the affinity for Na^+^ of the two transporters (Fig. 3E, F). Both transporters appeared to transport Na^+^ with low affinity. The half-saturation of the conductance was, however, observed at a 4-fold lower Na^+^ concentration in TdHKT1;4-1 than in TdHKT1;4-2 (*K*_M_ ∼3 and 12mM, respectively). These results also suggested that the maximal inward conductance was slightly (∼2-fold) lower in TdHKT1;4-1 than in TdHKT1;4-2 (Fig. 3E, F).

### Regulation of TdHKT1;4-mediated Na^+^ transport activity by external cations and osmolarity

**Fig. 4.**
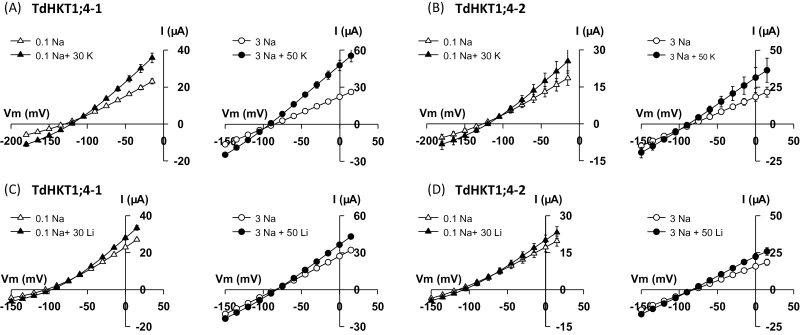
Effect of external K^+^ and Li^+^ on Na^+^ transport by TdHKT1;4-1 (A, C) and TdHKT1;4-2 (B, D). Bath solutions contained 0.1mM (left panels) or 3mM (right panels) Na^+^ (glutamate salts). They were or were not supplemented with KCl (A, B) or LiCl (C, D), with concentration of the added salt being 30mM (left panels) or 50mM (right panels). Data are means±SE (*n*=7 in A, *n*= 6 in B, *n*= 5 in C, *n*=8 in D) and are representative of two experiments performed on different oocyte batches.

Little is known so far on the regulation of HKT transporter activity. Na^+^ transport in several HKT transporters was reported to be sensitive to external K^+^ concentration, with K^+^ having an inhibitory effect (Gassmann *et al.*, 1996; Garciadeblás *et al.*, 2003; Jabnoune *et al.*, 2009; Mian *et al.*, 2011; Munns *et al.*, 2012). In particular, this is the case for TmHKT1;5 in *T. monococcum* at low (1mM) but not high (10mM) Na^+^ concentrations (Munns *et al.*, 2012). One report, in eucalyptus, also points to regulation of subfamily 1 HKT transport activity by osmolarity (Liu *et al.*, 2001). The effect of both external K^+^ and osmolarity was examined in the two durum wheat HKT1;4 transporters. The analysis of K^+^ effect on TdHKT1;4-mediated Na^+^ transport was performed at two (rather) low, external Na^+^ concentrations, 0.1 and 3mM (Fig. 4A, B). K^+^ was added at 30 or 50mM. Addition of KCl did not inhibit Na^+^ transport but, surprisingly, stimulated currents in both transporters (1.5- to 2-fold increase in conductance in the presence of K^+^; Fig. 4A, B). Stimulation of TdHKT1;4-1 and TdHKT1;4-2 activity by KCl concerned similarly inward and outward currents. It was accompanied by weak shifts of zero-current potential (5–10 mV in the presence of 0.1mM Na^+^ and 2–5 mV at 3mM Na^+^). This confirmed that K^+^ transport through the two TdHKT1;4 transporters was very limited, in agreement with selectivity analyses performed by external cation exchanges (Fig. 2), and therefore indicated that the stimulation of currents by KCl was essentially not due to K^+^ transport. As a comparison, the effect of another cation, Li^+^, on TdHKT1;4-1 and TdHKT1;4-2 activity was also examined (Fig. 4C, D). A similar stimulation of inward and outward currents was observed upon LiCl addition, although it was weaker. Thus, the observed stimulation of Na^+^ transport seemed weakly cation specific, and may reflect an effect of ionic strength on the activity of the two transporters.

The effect of osmolarity on TdHKT1;4-1 and TdHKT1;4-2 activity was examined in bath solutions containing 1mM Na^+^ by either decreasing the usual osmolarity by ~30% or increasing it ~2-fold. This led to a very slight decrease (by ~10%) of the inward and outward Na^+^ conductance in TdHKT1;4-1 and was without any effect in TdHKT1;4-2 (*n*=5; not shown).

**Fig. 5.**
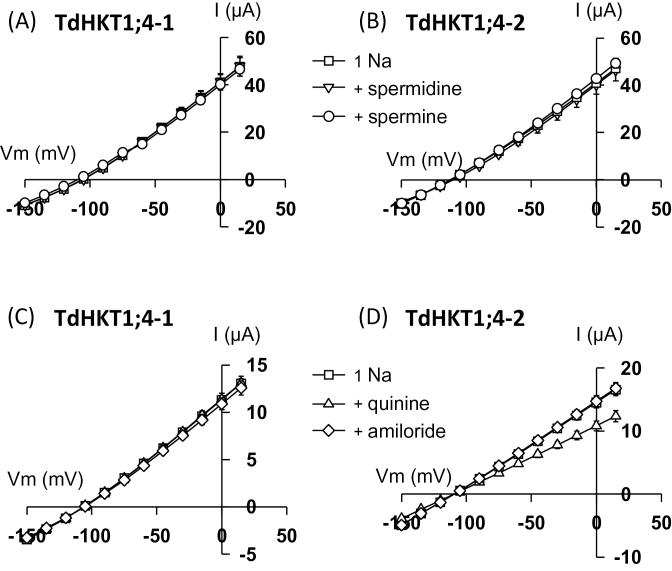
Effect of potential inhibitors on Na^+^ transport activity in TdHKT1;4-1 (A, C) and TdHKT1;4-2 (B, D). Control (1 Na) external solution contained 1mM Na^+^ as glutamate salt. Currents were recorded successively in the control solution and in the same solution but supplemented with 1mM spermine or 1mM spermidine (A, B) or with 500 µM amiloride or 500 µM quinine (C, D). Data are means±SE (*n*=7 in A, *n*=3 in B, *n*=5 in C and D).

Available analyses relative to ionic regulation of Na^+^-permeable systems mostly concern *in planta* experiments either performed on Na^+^ fluxes or on Na^+^/cationic currents across cell membranes recorded in patch-clamp experiments. Systems operating in these experiments, not molecularly identified, may correspond for some of them to HKT transporters. Polyamines, which have been shown to be involved in tolerance to abiotic stress (and in particular drought and salt stress), are known to modulate the activity of several plant ion channels, including Na^+^-permeable conductances (Shabala *et al.*, 2007; Zhao *et al.*, 2007; Alcázar *et al.*, 2010). In barley root epidermal cells, for instance, Na^+^ currents were reported to be strongly inhibited by spermidine and spermine added extracellularly at 1mM (Zhao *et al.*, 2007). The effect of spermidine and spermine was tested on the two wheat HKT1;4 transporters. Addition of 1mM spermidine or spermine to the bath solution containing 1mM Na^+^ did not affect TdHKT1;4 inward or outward currents (Fig. 5A, B).

*In vivo* analyses have also frequently mentioned several inorganic divalents (Ba^2+^, Ca^2+^, Zn^2+^), trivalents (La^3+^, Gd^3+^), and the quinine alkaloid as inhibitors of Na^+^ influx into tissues or of Na^+^ and/or non-selective cation currents across plasma membranes (Davenport and Tester, 2000; Demidchik and Tester, 2002; Zhao *et al.*, 2007; Schulze *et al.*, 2012). Based on these reports, the effects of Zn^2+^, La^3+^, and Gd^3+^ (at 100 µM) and quinine (at 0.5mM) were examined on TdHKT1;4-mediated Na^+^ currents in bath solutions containing 1mM Na^+^. The effect of amiloride, a classical inhibitor of Na^+^-coupled systems, was also examined at 0.5mM. Addition of Zn^2+^, La^3+^, Gd^3+^, or amiloride did not produce any effect on TdHKT1;4 currents (*n*=4, data not shown, and Fig. 5C, D). Quinine, in contrast, decreased (by ∼25%) TdHKT1;4-2 (but not TdHKT1;4-1) inward and outward currents (Fig. 5C, D). This suggests that some of the Na^+^ conductances already identified *in vivo* may correspond to HKT transporters and encourages further examination of quinine sensitivity within the HKT family.

## Discussion

### HKT1;4, a new functionally characterized HKT type

The cereal HKT family is thought, from rice knowledge and available information in other species, to possess six main genes, corresponding, based on rice nomenclature, to *HKT1;1/HKT1;2*-type, *HKT1;3*-type, *HKT1;4*-type, *HKT1;5*-type, *HKT2;1*/*HKT2;2*-type, and *HKT2;3*/*HKT2;4*-type (Garciadeblás *et al.*, 2003; Huang *et al.*, 2008; Hauser and Horie, 2010; Fig. 1). The transporters encoded by these main genes have almost all been functionally characterized in rice, and for two of them also in barley and/or wheat (Rubio *et al.*, 1995; Horie *et al.*, 2001; Garciadeblás *et al.*, 2003; Haro *et al.*, 2005; Ren *et al.*, 2005; Jabnoune *et al.*, 2009; Horie *et al.*, 2011; Mian *et al.*, 2011; Munns *et al.*, 2012; Oomen *et al.*, 2012; Sassi *et al.*, 2012). Only HKT1;4 type was not yet functionally characterized in any plant species. In rice, HKT1;4 displays 34–42% identity with other HKT members, its closest relative being HKT1;5.

Here, we identified and functionally characterized two HKT1;4-type transporters from durum wheat. The two transporters were expressed in *Xenopus* oocytes. In this expression system, high activity levels, comparable to those obtained upon expression of plant K^+^ or anion channels (which have been successfully characterized), were achieved for both transporters (Becker *et al.*, 2004; Geiger *et al.*, 2009; Jeanguenin *et al.*, 2011; Fig. 3). Thus, currents through these transporters could be recorded with good reliability, even in the presence of low concentrations of the main permeant cation (e.g. 100 µM Na^+^) or of weakly permeant cations: in all tested conditions, the macroscopic conductance of either transporter was at least five times higher than that of the endogenous systems of the corresponding control oocytes (Figs 2 and 3A, B), enabling precise determination of the transporter functional properties.

Cation exchange experiments and analysis of zero-current potentials in bath solutions varying in Na^+^ concentrations indicated that both transporters mediate passive preferential Na^+^ transport, and that the level of discrimination between Na^+^ and the other monovalent cations is the same in both transporters (Figs 2 and 3C, D). Estimation of ion permeability ratios in the two durum wheat HKT1;4 transporters using the Goldman–Hodgkin–Katz equation (as classically done in systems mediating diffusive transport of monovalents) gave *P*_X_/*P*_Na_=0.08–0.14 in both transporters, with X being K, Rb, Li, or Cs. This value is close to that determined for the rice HKT1;3 transporter (*P*_X_/*P*_Na_=0.06, with X being K, Rb, Li, or Cs; Jabnoune *et al.*, 2009; Sassi *et al.*, 2012). Less difference between Na^+^ and the other cations was observed for conductance (*G*_X_/*G*_Na_=0.25–0.3 in the two durum wheat transporters), as reported previously in the rice HKT1;3 transporter. Thus, the two TdHKT1;4 transporters are clearly Na^+^-selective systems, as expected for all subfamily 1 HKT members (Platten *et al.*, 2006). Interestingly, OsHKT1;3, like the two durum wheat HKT1;4 transporters, did not display discrimination between K^+^, Rb^+^, Li^+^, and Cs^+^ (Jabnoune *et al.*, 2009). This feature and a quite fixed value of permeability ratio between these cations and Na^+^ (close to 0.1) may be a trait of subfamily 1 members. Subfamily 2 HKT transporters, in contrast, appear more variable in their cation selectivity, showing major permeability differences among cations and between HKT members (Jabnoune *et al.*, 2009; Mian *et al.*, 2011; Sassi *et al.*, 2012).

In contrast to ion selectivity, Na^+^ transport affinity has been shown to be strongly variable within members of HKT subfamily 1: OsHKT1;1 displayed very low transport affinity when expressed in yeast or oocytes (e.g. half maximal conductance in oocytes at ∼75mM Na^+^), OsHKT1;3 transported Na^+^ also with low affinity, although with a 20 times lower *K*_M_ (half maximal conductance in oocytes at ∼3.5mM Na^+^), and HKT1;5 in rice and wheat displayed high-affinity Na^+^ transport (*K*_M_ in oocytes <1mM Na^+^) (Garciadeblás *et al.*, 2003; Ren *et al.*, 2005; Jabnoune *et al.*, 2009; Munns *et al.*, 2012). Na^+^ transport affinity of the two durum wheat HKT1;4 transporters characterized in the present study was low, similar to that of OsHKT1;3 for TdHKT1;4-1, and in between that of OsHKT1;1 (six times higher) and that of OsHKT1;3 (3.5 times lower) for TdHKT1;4-2 (Fig. 3E, F). Thus, characterization of the two durum wheat HKT1;4 transporters confirmed the high level of variability in Na^+^ transport affinity within cereal HKT subfamily 1. It is worth noting that large differences in conductance are also observed among the different HKT members when characterized in oocytes in similar conditions (Fig. 3; Jabnoune *et al.*, 2009; Munns *et al.*, 2012). For instance, the maximal conductances of TdHKT1;4-1 and TdHKT1;4-2 can be estimated to be about four and seven times larger, respectively, than the maximal conductance of the high-affinity TmHKT1;5 transporter (Fig. 3; Munns *et al.*, 2012). Although the expression levels of the different transporters are unknown in the plant, such differences in affinity for Na^+^ and maximal conductance strongly suggest that the different subfamily 1 HKT transporters in cereals have distinct physiological roles.

Direction of transport and level of inhibition by K^+^, have been identified as other sources of variability among cereal subfamily 1 HKT transporters. OsHKT1;1 displayed strong inward rectification in oocytes. The two durum wheat HKT1;4 transporters, like HKT1;5 and HKT1;3 in wheat and/or rice, allowed both inward and outward Na^+^ transport (Ren *et al.*, 2005; Jabnoune *et al.*, 2009; Munns *et al.*, 2012; Fig. 3A, B). Concerning the sensitivity to external K^+^, TdHKT1;4 transporters were, like OsHKT1;3 and OsHKT1;5, not inhibited by physiological concentrations of K^+^, in contrast to OsHKT1;1 and TmHKT1;5 (Ren *et al.*, 2005; Jabnoune *et al.*, 2009; Munns *et al.*, 2012; Fig. 4A, B).

As a whole, the characterization of the two durum wheat HKT1;4 transporters showed functional similarities with other HKT types of the cereal subfamily 1 but also differences with all other subfamily 1 members, indicating that none of the four main HKT types in cereal subfamily 1 are functionally redundant. Their functional diversity, notably in terms of Na^+^ transport affinity and sensibility to K^+^, may help the plant to tune its cellular Na^+^ fluxes to varying Na^+^ and K^+^ levels in different tissues and/or environmental situations.

### Diversity among wheat HKT1;4 transporters

In rice, exploitation of allelic variations between salt-tolerant and salt-sensitive ecotypes has led to the identification of a number of QTLs of plant salt tolerance, one of which is associated with the *HKT1;5* gene (Ren *et al.*, 2005). In *OsHKT1;5*, four non-synonymous nucleotide substitutions in the coding region differentiate the ‘salt-tolerant’ allele found in Nona Bokra from the ‘salt-sensitive’ one in Koshihikari (Ren *et al.*, 2005). Analysed in *Xenopus* oocytes, the Nona Bokra and Koshihikari OsHKT1;5 transporters slightly differed in Na^+^ transport ability (50% higher conductance upon expression of the Nona Bokra transporter), which could underlie the functional difference between the two alleles *in planta* (Ren *et al.*, 2005). In *Arabidopsis*, ecotypic variation in the *AtHKT1* gene could also be related to different leaf Na^+^ contents (Rus *et al.*, 2006). In this latter study, different levels of expression of *AtHKT1* could be linked to sequence differences in the *AtHKT1* promoter region.

In wheat species, different associations of genomes are an important source of salt tolerance variability. The D genome, which exists in the hexaploid bread wheat but not in the tetraploid durum wheat, for instance, has been shown to carry an essential salt tolerance locus that coincides with an *HKT1;5* gene (Gorham *et al.*, 1987; Dubcovsky *et al.*, 1996; Byrt *et al.*, 2007). Another salt tolerance locus coinciding with an *HKT1;5* gene was identified in the A genome of the wheat ancestor *T. monococcum* (Byrt *et al.*, 2007; Munns *et al.*, 2012). This locus is absent from durum or bread wheat, which do not possess *HKT1;5* genes in their A genome (Huang *et al.*, 2008). *HKT1;5*-*A* from *T. monococcum* was recently isolated and functionally characterized (Munns *et al.*, 2012). No *HKT1;5* gene from durum or bread wheat, out of the three expected in the B genome and that in bread wheat D genome (Huang *et al.*, 2008), were, however, characterized, preventing us getting clues on the reasons for their varying contribution to plant salt tolerance.

Variability in *HKT1;4* gene equipment in Triticae has also been associated with differences in plant salt tolerance (Huang *et al.*, 2006; James *et al.*, 2006, 2011). One of the two *HKT1;4* genes from *T. monococcum*, introduced through crossing in durum or bread wheat, was shown to improve Na^+^ exclusion from the blades upon salt stress (James *et al.*, 2006, 2011). Durum wheat is expected (from Southern blot analyses) to possess five *HKT1;4*-like genes: two in the A genome and three in the B one (Huang *et al.*, 2008). The two durum wheat HKT1;4 transporters identified in the present study were 89% identical. This not an extremely high degree of identity, and the fact that we could isolate the same two cDNAs in different durum wheat cultivars (data not shown) suggests that the two transporters are encoded by different *HKT1;4*-type genes rather than by two alleles of the same gene. Functional characterization of the two identified durum wheat HKT1;4 transporters showed a high similarity in selectivity but important differences in macroscopic conductances: 4-fold lower affinity for Na^+^ and 2-fold higher maximal conductance in HKT1;4-2 compared with HKT1;4-1 (Fig. 3E, F). These functional differences between the two durum wheat HKT1;4 transporters, which are stronger than those observed between the Nona Bokra and Koshihikari OsHKT1;5 transporters associated with a trait of salt tolerance (Ren *et al.*, 2005), are likely to be physiologically relevant. Thus, our study shows that important functional variability exists among durum wheat *HKT1;4* genes and encourages further examination of functional diversity among these genes in durum and other wheat species to better understand the basis of *HKT1;4*-related wheat variability in salt tolerance.

## Abbreviations:

HKT: high-affinity K^+^ transporter
I–V: current–voltage
QTL: quantitative trait locus
SE: standard error.
